# Amalgam

**Published:** 1904-03-15

**Authors:** H. P. Martin


### THE DENTAL REGISTER.

Vol. LVIII. MARCH 15, 1904. No. 3.

### AMALGAM.

BY H. P. MARTIN, D.D.S.

Read before the Detroit Dental Society, February 11, 1904.

In presenting a paper on such an old subject as amalgam, I can hardly expect to bring forth anything new. Should my feeble effort not be up to the standard, I trust it may bring out something in general discussion that will prove a great benefit to us all.

Having made no scientific study of the subject, I shall only attempt to deal with amalgam in a general practical way.

The advent of amalgam into dentistry dates back to 1826 and 1830. It was about the year 1830 that it was first used in America, and very crude filling material it proved to be. In fact, the dentist who dared to use it was not considered to be worthy of the name of dentist.

A great many improvements have been made in the composition and manipulation of amalgam since its first introduction into dentistry, for which we are greatly indebted to such men as Townsend, Chase, Flagg, Black and others.

The objectionable features of amalgam are color and change of form in setting. Some amalgams expand, others contract and sometimes they do both before they fully harden.

Contraction and expansion of amalgams is in a general way in harmony with the contraction and expansion of the metals of which they are composed, but as a filling material, contraction causes a cupping or pulling away from the walls of the cavity, while expansion causes a bulging from the cavity. The alloys which have an excess of tin contract a very great deal during the process of setting, while those alloys which contain from 70 to 80 percent of silver expand very considerably during the setting stage.

Black, in his experiments, found that an amalgam containing 65 percent silver and 35 percent tin produced the minimum amount of contraction and expansion and was most evenly balanced. Reducing the amount of silver and increasing the amount of tin produced a contracting amalgam; while increasing the amount of silver and reducing the amount of tin produces an expanding amalgam. Methods of manipulation influence results. An excess of mercury has much to do with its expansion or contraction. In mixing any amalgam we should have just enough of mercury to make the amalgam workable.

Black says that the cut of the alloy has a great deal to do with its contraction and expansion. The fine cut amalgam takes up a great deal more mercury and therefore causes the amalgam to contract because of its separation from the alloy in hardening. The evenness with which the mercury is distributed through the mass is a great factor in producing a good filling. A newly-cut alloy sets quicker than one that has aged. And alloys that make good fillings when freshly cut make bad ones when the alloy is aged. The flow or change of mass is a very objectionable feature to be found in all of the cheap or low-grade alloys. The constant pressure imposed upon the filling during mastication causes it to change form very considerably and in time secondary decay sets in.

The best results are to be obtained from a high-grade quick-setting alloy, which will give, I might say, permanent results in the hands of a skillful operator.

An ideal alloy would be one in which there is no perceptible contraction or expansion and which we could obtain from our dealers in the best form for use.

A good amalgam should be free from flow, should not contract or expand, and when set its crushing stress should be high. It should make a smooth, plastic mass and be capable of taking a high polish and not tarnish.

In preparing the cavity for amalgam every detail should be as carefully carried out as if a gold filling were to be inserted. Probably one great cause of failure is due to faulty preparation of the cavity. All cavities should be prepared somewhat on the lines I will enumerate.

All imperfect grooves or fissures, overhanging borders or margins should be cut away so as to make possible a perfectly-smooth finish to the filling.

In approximal cavities the buccal and lingual margins should be cut away sufficiently to expose the filling on either side. The cervical border of the cavity should be located below the free margin of the gum for protection, and the flow of the cavity should be flat, or at right angles to the tooth axis.

In approximal cavities of molars and bicuspids there should be a step cut in the occlusal surface and this should be but slightly dovetailed or grooved, as deep undercuts have a tendency to weaken the tooth, and are not necessary for anchorage.

In all disto or mesio-occlusal cavities a matrix should be used. In fact where there are not four walls to the cavity a matrix should be used, in order to obtain the best results, sufficient pressure must be used to make the filling one uniform mass, and where a wall is missing that cannot be accomplished without a matrix.

As to the sort of matrix to be used it is of little importance so long as it fits accurately at the gingival margin and retains its proper place giving the desired form for producing normal contour and approximal contact.

When the cavity is ready to receive the filling select the instruments most suitable for the case, as large as the cavity will admit for proper manipulation and place them ready at hand, always being careful to have them large enough to prevent chopping the amalgam and of such size as to condense the amalgam without bridging. Experience will teach us how best to incorporate the mercury with the alloy, as most every alloy has its peculiarity in this respect. Put the alloy in the palm of the hand and add sufficient mercury while triturating with the thumb to make a proper mixture, being careful not to use too much mercury, as an excess or an insufficient quantity will produce poor results in either case. Experimentation is the best means of finding out the exact proportions in which the alloy and mercury will combine. We should have the amalgam as dry as possible, consistent with easy working and complete combination.

Introduce into the cavity in as large pieces as cavity will permit and use pluggers that will not break it up into small particles, using sufficient pressure to make it a homogeneous mass when completed and to insure adaptation to the cavity walls.

If a quick-setting alloy has been used it is only necessary to wait a few minutes until it is hard enough to trim and polish.

In conclusion, I would say that amalgam should be classed among our standard materials for filling, as it has proper uses and is probably used more than any other material. Its sphere of usefulness covers a wide field in dental practice, in which, we, as yet, have found nothing to supplant it. In fact, I think I am safe in saying, that it ought to be classed as a permanent filling, which it is in the hands of skillful operators. In my judgment, all agree that a large percentage of failures is due to the operator who has not observed the first principle of proper cavity preparation, or used an alloy that is not fit to be inserted any mouth.

DISCUSSION.

DR. F. W. CRYDERMAN: The use of amalgam as a filling material has not been favorably thought of by the profession. From the very start it got a bad name because of the inferiority of the alloy and the class of practitioners who exploited it in an unprofessional manner for purely commercial reasons. Then it has encountered a great variety of vicissitudes in its use from the first till now. The work of Dr. Flagg, Palmer, Chase and Black, have to some extent redeemed it, until at the present time we have fairly good alloys and more definite results than ever before. Flagg made a strenuous fight to redeem the reputation of amalgam. Even going so far as to say that it was a better filling material than gold; especially for teeth that needed filling badly. He made what he called a "submarine" alloy which was an excellent tooth-saver, but unsightly, probably because it contained a large percent of copper. The dental profession has tried to imitate the medical profession and take a stand against all proprietary remedies and perhaps this may be a cause for the prejudice against amalgams whose alloys are manufacturers, secrets. We may think that because a manufacturer claims that he has a large percentage of some one or more of the precious metals in his alloy and he asks a large price for it, that it is more professional to use it. In fact some of these so-called high-grade, alloys contain little or no gold or platinum and are really the poorest of tooth-savers. Most of the alloys are made on the basis of 60 of silver to 40 of tin, The better alloys contain nearer 65 of silver and 35 of tin. Or in some cases about 5 percent of a copper and zinc alloy in place of that amount of silver and tin. Dentists would do better to make their own amalgams than to buy the cheap alloys they sometimes use. The process requires care but is not difficult. Use a good sized crucible and line it well with borax. Melt the tin and then add the silver, rolled thin and cut into small pieces, a little at a time, when these are fused add the copper prepared in the same way as the silver and then the zinc. The alloy should be stirred or shaken to secure good mixing. Some use powdered charcoal over the melted mass to prevent oxidation, others a stream of illuminating gas to take up the oxygen.

The casting of the ingot and cutting and ageing are important factors and should receive careful attention. In regard to the cavity preparation I would say that all cavities should be prepared to take advantage of the tendency of alloys to assume a spheroidal shape, round cavities, secure anchorage, and attenuated marginal borders will be found useful principles to be kept in mind.

DR. M. L. WARD: The makings of amalgams that will give the most satisfactory results is not such a simple process that any one can do it and get the best results. To begin with a suitable formula is essential. The so-called gold and platina alloys are mythical as they never contain more than 5 percent of these metals although some of them claim 15 percent. In fact these metals do not seem to have a practical value in alloys anyway. The silver, tin, copper and zinc alloys are almost universally used by the best manufacturers. And these alloys test and wear better than any others. They are the quick setting-alloys. Manufacturers experience great difficulty in securing pure metals, and consequently a well-organized formula will not always turn out uniform results. Every batch of metal must be tested, and its product always tested also before the formula can be relied upon. If the product is not up to standard it must be made so by varying the formula. Then the alloy must be cut and aged before it is put on the market, else the results will not be satisfactory. This is one of the reasons why amalgam has been so unsatisfactory as a filling material in the past. The manufacturers have not given sufficient care to the process of manufacture. There is a danger now that in the competition of manufacturers to sell their goods and to cheapen the selling price that good alloys will not be made with sufficient care to secure the best results. It is therefore not desirable that dentists undertake the manufacture of their own amalgam, neither is it desirable that they buy that made by dealers who know nothing of the proper process, or at least will not take the care to produce a uniformly reliable article. Out of a hundred amalgams on the market to-day, it is a conservative estimate that not over ten of them are properly made, or will give satisfactory results.

